# Do No Harm: A Review of Social Harms Associated with HIV Partner Notification

**DOI:** 10.9745/GHSP-D-23-00189

**Published:** 2023-12-22

**Authors:** Dawn Greensides, Kristina Bishop, Liz Manfredini, Vincent Wong

**Affiliations:** a Global Health Technical Assistance and Mission Support Project, supporting the Office of HIV/AIDS, Prevention, Care, and Treatment Division, U.S. Agency for International Development, Washington, DC, USA.; b U.S. Agency for International Development, Washington, DC, USA.; c Global Health Training, Advisory, and Support Contract, Credence Management Solutions, LLC, supporting the Office of HIV/AIDS, Prevention Care and Treatment Division, U.S. Agency for International Development, Washington, DC, USA.

## Abstract

**Introduction:**

HIV partner notification services (PNS) are highly effective in identifying people living with HIV, but the complex risks and repercussions associated with HIV testing and disclosure must be examined. The benefits of assisting people to learn their HIV status and link with treatment or prevention based on their results must be considered alongside the risks of adverse events or social harms.

**Methods:**

The study team systematically searched the PubMed, EBSCO, and Web of Science electronic databases (2015–2021), as well as abstracts from the International AIDS Society Conference (2016–2020) and the Conference on Retroviruses and Opportunistic Infections (2015–2020). Fifteen studies and 1 conference abstract met the inclusion criteria for this narrative review.

**Results and Discussion:**

Incidence of social harms across studies ranged from 0%–6.3%, with the higher range occurring among women with a previous history of intimate partner violence (IPV). The majority of the studies (69%) reported that less than or equal to 1% of participants experienced social harms as a result of PNS. Social harms included relationship dissolution, IPV, and loss of financial support.

**Conclusions:**

Evidence from a limited set of studies suggests that although social harms associated with HIV partner notification do occur, they are rare. Considering the rapid global scale-up of PNS, additional research and oversight are needed to provide countries with recommended minimum standards to support providers, clients, and their partners with safe partner notification.

## Abstract (precis)

This narrative review provides an in-depth interpretation of the limited evidence available on social harms associated with HIV partner notification services. Findings reflect knowledge gaps and areas where future research could make contributions.

## Teaser

**Key Findings:**

- The majority of studies reviewed reported less than 1% of participants experienced social harms as a result of HIV partner notification services.
- The most prevalent form of social harm presented across the studies was relationship dissolution, followed by violence and loss of financial support.

**Key Implications:**

- HIV partner notification services, using passive or assisted approaches, are a safe and effective method of identifying people living with HIV and linking them to care.
- Although social harms associated with HIV partner notification do occur, they are rare.
- It is important for countries to ensure the required minimum standards are in place to support providers, clients, and their partners without imposing unacceptable risks.

## INTRODUCTION

In 2022, the Joint United Nations Programme on HIV and AIDS (UNAIDS) estimated that there were 39 million people living with HIV (PLHIV) globally and that 86% of these individuals knew their status.^1^ To support reaching the UNAIDS global 95-95-95 HIV targets (95% of PLHIV know their status, 95% of those are on treatment, and 95% of those are virally suppressed by 2030)—particularly the first target of diagnosing 95% of all PLHIV—approaches that increase HIV testing and case finding need to be scaled up.

Evidence shows that partner notification, also known as index testing, is highly effective in identifying PLHIV.^2^^–^^6^ HIV partner notification services (PNS) is a focused approach in which the family members (including spouse and children) and sexual and/or drug-injecting partners of PLHIV are offered voluntary HIV testing services (HTS).^7^^,^^8^ This voluntary process involves a trained health care provider inviting a PLHIV to disclose the name(s) and contact information of their sexual and/or drug-injecting partners and of their biological children. If the HIV-positive client agrees, the provider extends the offer of PNS, in which the provider and client work together to determine which of the 4 World Health Organization-recommended referral approaches (Box) is most appropriate for contacting partners to offer them HTS. Per WHO guidelines, all PNS must adhere to the “5Cs of HTS”: consent, confidentiality, counseling, correct test results, and connection to HIV prevention (for both HIV-positive and HIV-negative individuals), care, and treatment (for HIV-positive individuals), and must also include intimate partner violence (IPV) screening and connection to services.^7^^,^^8^

BOX Methods for Partner Notification Services or Index Testing

**Patient referral (passive):** When a client discloses their HIV status to a partner and encourages the partner to go for HIV testing services (HTS).

**Provider:** When the provider notifies the contact, with the consent of the index client and offers the partner(s) voluntary HTS.

**Contract (delayed):** When an HIV-positive client enters into a contract with the provider to disclose to their partner(s) and suggest HTS within a mutually agreed upon time frame. If the partner(s) do not access HTS or contact the provider within that period, the provider will contact the partner(s) directly to offer voluntary HTS.

**Dual referral:** When a trained provider accompanies and provides support to HIV-positive clients when they disclose their status and the potential exposure to HIV to their partners. The provider then offers voluntary HTS to the partner(s).

The WHO's PNS guidelines published in 2016 highlighted the high potential yet low coverage of PNS.^7^ The 2016 guidelines urged implementers to offer PNS as part of a comprehensive package of testing and care offered to people with HIV. The 2016 guidance also reported that instances of social harm and other adverse events following PNS were rare.^3^^,^^7^ A separate study funded by the U.S. President's Emergency Plan for AIDS Relief and conducted by the U.S. Centers for Disease Control and Prevention examined index testing across 20 countries between 2016 and 2018. The study showed that as index testing scaled up, rates of positive HIV tests increased 64% among persons aged 15 years and older (from 7.6% to 12.5% positivity) and 67% among persons aged younger than 15 years (from 1.2% to 2.0% positivity).^6^ Based on the expected low risks of social harm and high potential for HIV testing positivity, the U.S. President's Emergency Plan for AIDS Relief, as well as a number of ministries of health, in line with WHO standards, also recommended the scale-up of index testing to increase case finding.^9^^,^^10^

However, as countries move to scale up PNS, concerns from advocacy and civil society organizations indicate that it is important to examine the complex risks and repercussions associated with HIV testing and disclosure, particularly for key populations and adolescent and young women.^11^^–^^13^ The benefit of assisting people to learn their HIV status and link with treatment or prevention must also be considered alongside the risk of an individual experiencing an adverse event or social harm. With that in mind, we undertook a review of the literature to identify the frequency of adverse events and social harms associated with HIV PNS. Information gathered from this review will inform development of further guidance to improve PNS approaches to ensure all individuals accessing these services are protected and the risk of adverse events or social harms associated with PNS is minimized.

The benefit of assisting people to learn their HIV status and link with treatment or prevention must also be considered alongside the risk of an individual experiencing an adverse event or social harm.

## METHODS

### Literature Search

The study team conducted an electronic database search of PubMed, EBSCO (Elton B. Stevens Company), and Web of Science for articles published in 2015–2021. The team also searched for abstracts available from the International AIDS Society Conference (2016–2020) and the Conference on Retroviruses and Opportunistic Infections (2015–2020). For the electronic database, conference abstract, and gray literature search, the title and abstract search was conducted using the following terms: “index testing” or “partner notification” and “HIV” combined with 1 or more of the terms “key populations,” female sex worker (“FSW”), men who have sex with men (“MSM”), injection drug use (“IDU”), “prison/incarcerated populations,” “pregnant women,” “antenatal,” gender-based violence (“GBV“), “IPV,” “abuse,” “coercion,” “forced,” “unauthorized,” “threats,” “blackmail,” “violations,” “ridicule,” “harassment,” “adverse events,” “social harm,” “unanticipated events,” and “consequences.”

Each abstract was reviewed independently for its relevance to the topic by 1 researcher and verified by a second researcher. Abstracts determined irrelevant were discarded from the review. Relevant abstracts were selected for independent full-article review by 2 researchers. Each article that underwent full-text review was also assessed for additional relevant references, which were also reviewed.

### Inclusion Criteria

Literature was included if the article discussed PNS and/or index testing, assessed associated adverse events or social harms, was published between 2010 and 2021, and was written in English (Figure).

**FIGURE:**
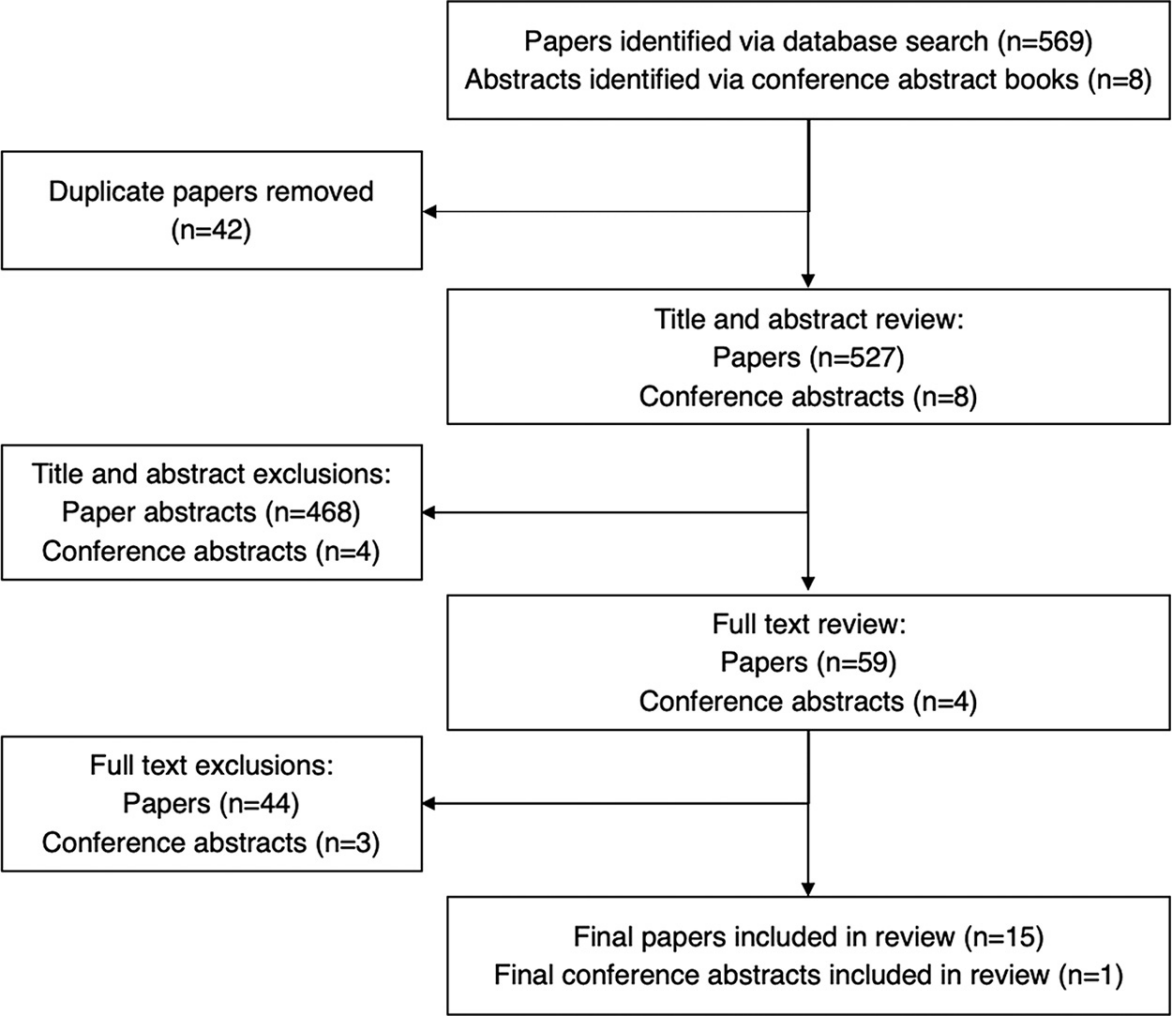
Literature Search Flow Diagram

### Data Abstraction

Data extraction and content analysis were conducted by 1 researcher and verified by a second study team member. Abstraction categories include the study description, sample characteristics, detailed findings, description of the adverse event(s)/social harms, incidence of adverse event(s)/social harms, and study conclusions.

### Data Synthesis

The content of all included articles and abstracts was analyzed to identify themes on social harms associated with PNS with the goal of expanding and deepening the evidence base and identifying new areas to address within this topic area.

A meta-analysis was not performed due to a number of factors, including the lack of a consistent definition of adverse events or social harms across included studies; the measurement of adverse events and social harms as a secondary finding of the study; and the low incidence of adverse events or social harms, which reduced the study team's ability to explore differences between individuals experiencing them. Instead, a narrative review was performed.

For this review, adverse events and social harms described in the reviewed studies included violence (IPV, gender-based violence, sexual violence, physical violence, and psychological violence), relationship dissolution, loss of financial support, deprivation, emotional abuse, and legal harm.

## RESULTS

Of the 569 articles and 8 conference abstracts identified, 59 articles and 4 conference abstracts met the inclusion criteria for this review, but only 16 articles and 1 abstract presented quantitative data on social harms after PNS (Table).^2^^,^^4^^,^^5^^,^^14^^–^^27^ Of the 16 articles that presented quantitative data, 1 was not included in the analysis,^14^ as it was data from exploratory qualitative interviews asking women living with HIV retrospectively about their disclosure experience.

Among the 16 studies (15 articles and 1 conference abstract) reporting data on the incidence of social harms, 2 articles focused specifically on key populations, 2 focused on women who had recently experienced IPV, 2 focused on women in antenatal care settings, 1 study was in a refugee setting, and the remaining 9 studies focused on the general population. All but 1 of the studies (Vietnam) were in sub-Saharan Africa: 5 in Kenya; 2 each in Cameroon, Malawi, Tanzania, and Uganda; and 1 each in Mozambique and Zambia.

**TABLE.** Studies Included in Review of Adverse Events of Social Harm Associated with HIV Partner Notification Services

Author, Year	Country, Study Description, and Sample Characteristics	Index Clients Experiencing Social Harm, No. (%) and Type of Social Harm	Study Conclusions
Kahabuka et al., 2017^2^	Tanzania; cross-sectional study assessing the acceptability and measured effectiveness of PNS (passive, contract, provider); general population. Sample: 390 index clients (53% female).	0 (0) (The study did not actively follow index clients for IPV reporting.) No notification-related harms reported.	Partner notification could dramatically increase the number of previously undiagnosed PLHIV who learn their status and are linked to care.
Cherutich et al., 2017^4^	Kenya; cluster randomized control trial looking at safety and effectiveness of immediate versus delayed APNS; general population. Sample: 1,119 index clients (62% female).	2 (0.2) Type of harm not defined but determined to be related to passive notification not APNS.	APNS are safe and significantly increase HIV testing and case detection among partners of men and women testing HIV-positive in the clinical setting. Programs should consider screening clients to identify those at highest risk of IPV, counseling them, and referring them to specialized IPV management centers.
Tih et al., 2019^5^	Cameroon; review of programmatic findings of Cameroon Baptist Convention Health Services APNS program; general population. Sample: 976 index clients (no gender breakdown).	11 (1.1) IPV; 15 (1.5) loss of financial support; 61 (6.3) relationship dissolution.	The implementation of APNS as a large-scale public health program is feasible. APNS helped identify many PLHIV who did not know their status, who were then counseled on HIV prevention strategies if HIV-negative or referred to HIV care if HIV-positive. Social harms and IPV occurred infrequently after APNS.
Brown et al., 2011^15^	Malawi; comparison of patient referral, contract referral, and provider referral among clients with newly diagnosed HIV in an STI clinic setting; general population. Sample: 240 index clients (58% female).	2 (0.8) Relationship dissolution (n=1), police report (n=1).	Active partner notification was feasible, acceptable, and effective among STI clinic clients. Partner notification will increase early referral to care and facilitate risk reduction among high-risk uninfected partners.
Dijkstra et al., 2021^16^	Kenya; assess whether HPN services offered to GBMSM and TGW is acceptable, feasible, and safe; key populations. Sample: 17 index participants.	0 (0) No notification-related harms reported.	HPN services offered to GBMSM and TGW appeared acceptable, feasible, and safe.
Goyette et al., 2018^17^	Kenya; an analysis of a subset of index participants from Cherutich et al.^4^ to determine whether history of IPV modifies the effectiveness and risk of relationship dissolution associated with HIV APNS; high-risk women. Sample: 81 index clients who experienced physical or sexual IPV >1 month before enrollment.	2/1,119 (0.2) from Cherutich et al.^4^ APNS was not associated with physical or sexual IPV.	Among participants who had not experienced IPV in the last month but had experienced IPV in their lifetimes, the results suggest that APNS are an effective and safe partner notification strategy. Unrelated to APNS, those with a history of IPV had higher rates of relationship dissolution than those without a history of IPV.
Henley et al., 2013^18^	Cameroon; structured program evaluation of HIV partner services from ANC, voluntary counseling and testing, and inpatient facilities; general population. Sample: 1,462 index clients interviewed (73% female).	0 (0) No notification-related harms reported.	HIV partner services can be successfully implemented and are highly effective in identifying and linking people to care. Further investigation of the social harms and benefits associated with partner services is needed.
Kariithi et al., 2021^19^	Kenya; hybrid type 2 implementation science study to the effectiveness of APNS when integrated into routine HTS and evaluate the integration, implementation fidelity, acceptability, demand, and costs of the intervention; general population. This report is based on preliminary analyses. Sample: 1,724 index clients (100% female).	35 (2) Events were listed solely as “IPV,” including 32 relationship dissolutions.	APNS is an effective modality for reaching those who are undiagnosed. Final results of the study will help bridge the gap between clinical research findings and real-world practice and provide guidance regarding optimal strategies for APNS integration into routine HIV service delivery.
Klabbers et al., 2020^20^	Uganda; mixed-methods study to understand the role of interpersonal violence in APNS for HIV; refugees. Sample: 195 index clients (no gender breakdown).	8 (4) 7 of the 8 involved sexual partners for whom a prior history of IPV had been reported Interpersonal violence defined as physical, sexual, or psychological violence, or deprivation and neglect, perpetrated by partners, family, or community members.	Fear and occurrence of disclosure-related violence are intertwined with cultural perceptions and associations regarding HIV. Future research is needed to prospectively evaluate how prior experiences of violence affect APNS participation and to investigate whether APNS is associated with subsequent violence.
Mutale et al., 2021^21^	Zambia; 2 parallel randomized trials to address gaps in male partner testing: HIV-positive and HIV-negative pregnant women from ANC setting randomized to receive APNS or secondary distribution of HIV self-test kits; ANC attendees. Sample: 100 HIV-positive women; 200 HIV-negative women.	1 (0.3) IPV, male partner abandonment, emotional and legal harm.	Adding HIV self-testing to PNS can expand the coverage of male partner HIV testing and help to identify those in immediate need of HIV prevention or treatment. Strategies relying on secondary distribution of HIV self-test kits can place an undue burden on pregnant and breastfeeding women. Given the challenges inherent to HIV status disclosure, resources are needed to minimize IPV and social harms and actively support those who face such issues.
Myers et al., 2016^22^	Mozambique; pilot APNS program to assess acceptability, effectiveness, and safety in a large, urban clinic; general population. Sample: 206 index clients (79% female).	2 (1) Relationship dissolution (n=2) with loss of financial support (n=1). Additionally, none of the 173 partners interviewed reported experiencing social harm after their partner's diagnosis	APNS that relies on a community health worker labor force is acceptable to clients, safe, and effective in promoting HIV testing among the sex partners of newly diagnosed HIV-infected persons. While continued consideration of IPV is needed, this concern should not be an impediment to the scale-up of APNS.
Namimbi et al., 2020^23^	Uganda; examination of the acceptability and effectiveness of APNS services in 2 urban health facilities; general population. Sample: 35,704 index clients (65% female).	354 (1) Post-notification GBV, not further defined.	APNS is a feasible and efficient approach for identification of HIV-positive cases and should be integrated into routine clinical services. However, there is a need to monitor trends and strengthen GBV services.
Nguyen et al., 2019^24^	Vietnam; Implementation study to understand and assess the feasibility and effectiveness of implementing APNS as part of community testing services for key populations. Sample: 186 index clients (no gender breakdown).	0 (0) No notification-related harms reported.	As part of community-led HTS, APNS is an effective and feasible HIV case-finding strategy for key populations. It is critical that programs sensitize communities and health workers and prepare and train peer educators to provide the necessary support, messages, and tools to monitor and report social harm.
Plotkin et al., 2018^25^	Tanzania; mixed-methods, cross-sectional study on PNS to analyze differences in success of referral for HTS among male vs. female and between married and unmarried index clients; general population. Sample: 390 index clients (53% female).	4 (1) Relationship dissolution.	PNS integrated into routine facility HTS is an effective way to reach previously undiagnosed HIV-infected individuals. Formative studies of gender dynamics and gender-related barriers and facilitators of partner notification for men compared to women, including studies of both attitudes to and experiences with IPV, are recommended either before partner notification programs are rolled out or associated with roll-out.
Rosenberg et al., 2015^26^	Malawi; unblinded, randomized control trial to compare 2 strategies for recruiting male partners for couples HTS through ANC: invitation only versus invitation plus tracing; ANC attendees. Sample: 181 index clients (100% female).	3 (1.7) Relationship dissolution (n=2), emotional distress (n=1).	An invitation plus tracing strategy is highly effective at increasing couples' HTS uptake. Invitation plus tracing with couples' HTS could have many substantial benefits if brought to scale. Those concerned about partner anger, violence, or abandonment were less inclined to return with a partner. These findings suggest women can judge whether partner recruitment is safe.
Sharma et al., 2021^27^	Kenya; assessment of APNS acceptability, reasons, and predictors of non-enrollment among females in an ongoing implementation project of APNS scale up; general population. (Same study as Kariithi et al.^19^) Sample: 839 index clients (100% female).	0.7% IPV; 1.9% relationship dissolution. (Numerical data not provided).	APNS has high acceptability among HIV-positive females regardless of age or testing history and can be safely scaled up among female index clients.

Abbreviations: ANC, antenatal care; APNS, assisted partner notification services; GBMSM, gay, bisexual, and other men who have sex with men; GBV, gender-based violence; HPN, HIV partner notification; HTS, HIV testing services; IPV, intimate partner violence; MSM, men who have sex with men; PLHIV, people living with HIV; PNS, partner notification services; STI, sexually transmitted infection; TGW, transgender women.

The studies encompass 43,978 participants, who may include overlapping populations. The range of social harms reported across the studies included relationship dissolution, loss of financial support, and IPV (including physical violence, verbal abuse, threats, and sexual violence). Among the 16 studies that examined social harms associated with PNS, the range of social harms experienced ranged from 0% to 6.3%. Eleven studies reported that less than or equal to 1% of participants experienced social harms as a result of PNS, of which 4 studies reported no social harms were experienced. Two articles did not define the reported social harms. Included studies and associated findings are described in detail in the Table.

The most prevalent form of social harm presented across the studies was relationship dissolution, followed by violence (few of the studies detailed the type of violence experienced) and loss of financial support.

The most prevalent form of social harm presented across the studies was relationship dissolution, followed by violence and loss of financial support.

Eight studies reported relationship dissolution/partner abandonment ranging from 0.3% to 6.3%.^5^^,^^15^^,^^19^^,^^21^^,^^22^^,^^25^^–^^27^ Among these studies, the term “relationship dissolution” was used to convey the ending of a relationship by the voluntary activity of at least 1 partner, with no distinction as to which partner ended the relationship. The term “partner abandonment” was used only twice when the female index client reported being left by a male partner. Additionally, 2 studies reported loss of financial support.^5^^,^^22^ Of the 8 studies reporting relationship dissolution, 4 had both male and female index clients, yet relationship dissolution was reported only by female clients.^5^^,^^15^^,^^22^^,^^25^

Six studies reported that participants experienced partner violence after HIV status disclosure ranging from 0.3% to 6.3%,^5^^,^^19^^–^^21^^,^^23^^,^^27^ with the higher range occurring among women with a previous history of IPV. Three studies that assessed violence included only female index clients, with a range of 239–1,724 participants.^19^^,^^21^^,^^27^ Three studies included male and female index clients, with a range of 195–35,704 participants. These studies did not disaggregate instances of IPV by gender, which ranged from 1% to 4% across the studies.^5^^,^^20^^,^^23^ Of note, the mixed gender study with the highest rate of reported IPV (4%) noted that 88% of those involved sexual partners for whom a prior history of IPV had been reported.^20^

Twelve studies reported implementing IPV screens before PNS.^2^^,^^4^^,^^5^^,^^16^^–^^22^^,^^25^^,^^27^ Among these, 4 provided closer monitoring, counseling, and/or safety protocols for that index client during PNS.^4^^,^^17^^–^^19^ Four studies excluded subjects with a previous history of IPV within the past 30 days.^4^^,^^16^^,^^17^^,^^19^ Among the studies with IPV screening and risk mitigation procedures in place, IPV instances ranged from 0% to 6.3%.^2^^,^^4^^,^^5^^,^^16^^–^^22^^,^^25^^,^^27^ Of note, 1 of these studies specifically included only women who had experienced IPV within the previous year^17^; if this study is removed, the rates of IPV across the studies with IPV screening and risk mitigation measures in place falls to 0%–2%. Four studies examining IPV did not include pre-PNS IPV screens within their methodology.^15^^,^^23^^,^^24^^,^^26^ Instances of IPV among these studies ranged from 0% to 1.7%.

The types of PNS offered and provided in each of the studies varied, with 10 studies counseling index clients on passive referral, provider referral, and/or contract referral or dual referral.^2^^,^^5^^,^^15^^,^^16^^,^^18^^,^^20^^,^^21^^,^^24^^,^^25^^,^^27^ Three studies provided either immediate provider notification or delayed provider notification,^4^^,^^17^^,^^22^ 2 studies provided only provider notification,^19^^,^^23^ and 1 provided either a written invite or provider notification.^26^ Of the studies that provided the index client a choice of PNS options, only 1 reported social harm data per type of partner notification selected.^20^ Of the 8/195 cases that reported post-PNS IPV, 3 had provider notification and 5 had contract (delayed, but called “assisted PNS”) notification.

Overall, all of the studies looked at social harms resulting from disclosure of HIV status to sexual partners. No studies identified adverse events due to the health care system not meeting minimum standards (i.e., breaches of confidentiality, coercion, withholding treatment, or unauthorized disclosure). One study^22^ reported that even though the study protocol directed health care providers not to contact partners if the index client had a history of IPV, several providers did so. Despite this deviation from the protocol, the absence of social harms in this study is reassuring.

It is challenging to draw a direct relationship between HIV PNS and social harms given that newly diagnosed PLHIV do not live within the vacuum of their diagnosis and can experience other risk factors that may contribute to experiencing social harms. Defining the parameters of what an adverse event or social harm is also presents challenges. For example, relationship dissolution, which was among the more common experiences reported, may not be due to disclosure and may instead be an appropriate reaction to changing life circumstances.

Additionally, issues around confidentiality and coercion have been raised in the literature and by some members of civil society.^11^^,^^28^ HIV PNS attempts to get the contacts of PLHIV tested by empowering providers to support clients through the process, either actively or passively. Despite these concerns, findings from studies in this review are reassuring in that the measured social harms resulting from partner notification using passive or assisted approaches are rare. However, there was little information available in the literature on the long-term unintended consequences of PNS and social harm and on the consequences of PNS among key populations and adolescent and young women, in particular. Health care workers are positioned to provide flexible PNS options so that clients can make the best decision based upon their individual circumstances to help minimize the occurrence of associated social harms. The discussion section is framed around these topics.

## DISCUSSION

### Long-Term Unintended Consequences of PNS

Very few of the studies followed up index clients longer than 6 weeks after testing. Two studies followed up index clients at 6 weeks, 6 months, and 12 months^19^^,^^27^ but only discussed social harms reported at the 6-week follow-up, which included both IPV and relationship dissolution at rates lower than 2%. Future studies should assess index clients longitudinally to determine if there are any long-term risks associated with PNS that were not captured within the studies in this review. Additionally, legal provisions for protecting PLHIV against potential harm following PNS are lacking, particularly for marginalized and criminalized populations.^7^^,^^11^ Adverse events and social harms may go unreported in places where stigma and discrimination, violence, blackmail, extortion, and arrests go unchecked or where no structures exist for reporting the risk for or experiences of violence.^11^^,^^29^ This lack of structural guidance or established systems can contribute to the occurrence of adverse events and social harms with little to no accountability.^11^^,^^30^ Furthermore, the absence of health care provider motivation to report social harms and of current structures for reporting increase the risk that social harms will be under-reported and unaddressed.

Several of the included studies mentioned low incidence of IPV because women who experienced higher risk of IPV were either not eligible for PNS or likely elected not to participate in PNS.^4^^,^^18^^,^^21^ Exclusion of those who have reported a history of IPV is not stipulated in the WHO guidelines; rather, the guidelines recommend assessing risk of harm on a case-by-case basis in consultation with the index client.^7^ Integrating a flexible approach within PNS programs so that the index client can choose the safest option and so health care workers can adjust their monitoring approaches based upon each client's risk level may help to reduce risk.^31^ Rigorous monitoring of index clients who are at risk for IPV may further mitigate risk.^5^^,^^20^^,^^32^ However, even quality IPV screening can enable false negatives when clients are not comfortable disclosing incidents of violence with their provider. This is often due to insufficient training or incomplete understanding of the topic among providers.^33^ Index clients must be allowed the personal agency to decline PNS based on broader concerns, even if these are not specifically related to reported physical, emotional, or sexual violence.^2^^,^^26^^,^^27^^,^^34^ There was a wide range of social harms reported across the review, which prompts the need for further studies focusing on the frequency and severity of adverse events.^15^^,^^18^^,^^20^^,^^25^ Only 1 study looked at social harms perpetrated not just by the sexual partner but by extended family and/or community members.^20^

Integrating a flexible approach within PNS programs so that the index client can choose the safest option and so health care workers can adjust their monitoring approaches based upon each client's risk level may help to reduce risk of IPV.

Given the emphasis on PNS and the scientific evidence showing its potential for reaching and testing hard-to-reach populations, these potential, longer-term unintended consequences could have detrimental effects on epidemic control.

### Consequences Among Key Populations

Key populations worldwide commonly experience violations of privacy, breaches of confidentiality, and coercive medical practices outside of PNS.^11^^,^^30^^,^^35^ Given the rights violations that occur among key populations at baseline, these groups are at increased risk for any potentially deleterious long-term consequences of index testing that may exist. Also, although all marginalized groups share certain risks, specific subgroups (sex workers, people who inject drugs, men who have sex with men, transgender women, and prison populations) may encounter unique PNS-associated risks. Literature that specifically investigates the long-term consequences and risks of index testing on key populations and key population subgroups is sparse. Further research is critically necessary.

### Consequences Among Adolescents and Youth

Additionally, the dynamics of HTS and PNS for youth, especially adolescent girls and young women, need to be critically considered. Although a few studies in this review included index clients aged 15 years and younger,^18^^–^^20^^,^^23^^,^^24^^,^^26^^,^^27^ there was no distinction or consideration of rates of social harms across the different age groups. Unequal power dynamics between health care providers and youth, particularly adolescent girls and young women, may lead to coercion and unsafe PNS, and in many countries, youth aged younger than 18 years face restrictions on their decision-making.^12^^,^^13^ More research is needed to assess PNS preferences.

### Health Care Worker Capacity

Across the existing literature on the social harms associated with PNS, health care providers are critical to ensure adherence to ethical, safe guidelines.^34^^,^^36^^,^^37^ Providers must be well trained, sensitized, and able to tailor and adjust standard PNS guidelines to key and other marginalized populations. To ensure that index clients are informed and fully understand their personal risks and how to weigh these against the benefits, it is critical that providers clearly and consistently discuss the risks of PNS, even when partner confidentiality is maintained. This was highlighted in a qualitative study in Kenya in which participants reported inadequate discussion by providers—or no discussion at all—about the socially adverse outcomes of PNS, particularly the risk of partner violence. In addition, they reported receiving only “broad” advice from providers on how to disclose to partners safely. Most notably, those who experienced partner abuse post-disclosure said that they had received no counseling on how to disclose HIV status safely and no information on the risks of IPV post-disclosure.^14^

Index clients also need to hear carefully crafted, targeted, and culturally relevant educational messages about the benefits of PNS to make an informed choice about participating.^16^^,^^24^^–^^27^ Building and maintaining the trust of the community is arguably among the most important roles of health care providers. Harms that are derived from the health care system not meeting minimum standards (i.e., breaches of confidentiality, coercion, withholding treatment, and unauthorized disclosure) with PNS can serve as strong deterrents to seeking HIV testing, care, and treatment services.^7^^,^^11^^–^^13^ The experience of social harm can erode the trust between clients and HIV testing centers. If PNS are perceived as involuntary, people may become fearful of getting tested if they believe, perceive, or are told that they will be forced to share information about their sexual network. Therefore, implementing PNS without using the existing evidence-based, ethical, and rights-based WHO guidelines can directly threaten the success of HIV testing services.^7^

### Strengths

This narrative review provides a thoughtful and in-depth interpretation of the limited evidence available to extend the understanding of social harms associated with PNS. Findings reflect a holistic understanding of the subject, and the synthesis of the available evidence allowed for the identification of knowledge gaps and areas where future research could make significant contributions. By focusing on the social harms associated with PNS, this review not only highlights the immediate concerns but also offers a foundation for policymakers, practitioners, and implementers to address the challenges in a comprehensive manner.

### Limitations

This review has several limitations. It is important to note that this is not a meta-analysis and is inherently at risk for bias within the search methodology, though the authors took steps to minimize this. It is very challenging to identify direct causal associations between social harms reported in the studies and HIV PNS, given the complexity of experiences for an individual who is first learning their diagnosis. At present, most of the literature on the effectiveness and safety of PNS focuses on the general population. The relative dearth of data on the topic, contextual differences between countries, and the lack of available studies that include key populations raise concerns about the generalizability of the studies. There is a need to conduct additional safe, ethical research among key populations and youth on the potential for adverse events and social harms related to PNS. It is important to note that the majority of studies assessed for IPV but may not have identified other types of harms or adverse events related to the actual provision of partner-associated disclosure, including provider coercion, breaches in confidentiality, etc. Further limitations include the fact that many of the included studies often only followed up with a small subset of index cases who appeared for follow-up interviews. Index participants who did not have follow-up interviews may have experienced higher or lower rates of social harms that remained undocumented. In addition, most of the studies followed up between 4 and 8 weeks post-PNS. More longitudinal data are required to understand any long-term adverse effects of PNS that were not identified. There is no standard definition for a social harm or adverse event, which limited our ability to conduct a meta-analysis.

## CONCLUSIONS

Evidence suggests that HIV partner notification services are a safe and effective method of identifying PLHIV and linking them to care, but specific care must be taken to ensure clients who are at risk of IPV are appropriately counseled and referred to specialized services.

This study did not identify any instances of provider coercion in the literature. Considering the rapid global scale-up of index testing and PNS, additional research and oversight are needed to ensure minimum standards are in place to support providers, clients, and their partners without imposing unacceptable risks.

## Notes

**Peer Reviewed**

**First published online:** xxxx.
